# Positioning, power and agency in postgraduate primary care supervision: a study of trainee narratives

**DOI:** 10.1186/s12909-023-04826-9

**Published:** 2023-11-17

**Authors:** Dawn Jackson, Josephine Brady, Donna Dawkins

**Affiliations:** 1 College of Medical and Dental Sciences, University of Birmingham, Edgbaston, Birmingham, B15 2TT UK; 2 Mary Immaculate College, South Circular Road, Limerick, V94 VN26 Ireland; 3 School of Education, University of Birmingham, Edgbaston, Birmingham, B15 2TT UK

**Keywords:** Postgraduate, Supervision, Narrative

## Abstract

**Background:**

Postgraduate supervision takes place within complex training environments, where experiences are shaped by the socio-cultural context and wider profession, and where tensions permeate. Bordin’s working alliance-based model of supervision suggests that quality relationships encompass agreement on the goals and tasks of supervision, in the context of an emotional bond. However, as trainees and their supervisors navigate the demands of providing safe clinical care, alongside educational support, disagreement on expectations for supervision may emerge. By applying a critical lens, this research draws on positioning theory to explore General Practice trainees’ experiences of supervision.

**Methods:**

In 2017–2019 a series of narrative interviews were undertaken with 13 General Practice trainees in the United Kingdom (UK). Participants were purposively sampled based on end-of-year performance, gender, training location and training status. Interviews were analysed using Brown and Gilligan’s Listening Guide, which was adapted to incorporate an exploration of positioning, power and agency.

**Results:**

Trainees appeared to hold variable positions, such as ‘insiders’, ‘outsiders’, ‘peers’ and ‘problem trainees’. Supervisors, through talk and the degree of access afforded, contributed to this positioning. Some trainees viewed their supervisors as brokers and guides as they navigated their training, whilst others were suspicious of the supervisor role. For trainees who raised concerns about their supervisor through formal channels, results were not often satisfactory. Others chose to navigate difficulty in supervision through informal means. This typically involved mastery of artefacts of training, such as the electronic appointment book or training portfolio.

**Conclusions:**

This paper builds on Bordin’s model of supervision to encourage greater clarity in supervisory discussions, exploring assumptions, and recognising the influences of environment, power, positioning, and agency. We have developed a Model of the Supervisory Alliance in Postgraduate GP Training (MSA-GP) to serve as a springboard for discussion for trainees and their supervisors.

**Supplementary Information:**

The online version contains supplementary material available at 10.1186/s12909-023-04826-9.

## Background

Supervisory relationships are considered a key source of support for postgraduate clinical trainees in many international settings [1–3]. Yet competing goals and priorities must also be navigated in and through these relationships [4]. A balance must be struck between support for the trainee and encouragement of autonomous practice, alongside upholding patient safety, meeting training programme demands with respect to assessment and examinations and contributing to clinical workload commitments [4–8]. Tensions and competing interests have been considered as ubiquitous in medical supervision, and quality supervisory relationships are cited as one way to mediate these [9, 10]. Similar to the ‘therapeutic alliance’ between a client and their therapist in the field of counselling, the ‘educational alliance’ is considered to be foundational to the trainee’s progression and development [3, 11]. Bordin’s working alliance based model of supervision suggests that quality supervisory relationships encompass agreement on the goals and tasks of supervision, in the context of an emotional bond [4, 12].

However, within an environment of socio-cultural complexity and competing expectations, it is quite possible that trainees and their supervisors may disagree on their perceptions of the goals of supervision, or the tasks required to facilitate professional development. It has been suggested within the literature that supervisor and trainee perspectives on the alliance can diverge [13, 14]. In our previous research, which explored the perceptions of experienced GP supervisors, such stories of divergence were described [15]. The supervisors noted variable expectations between supervisors and trainees, and subsequent breakdown of the supervisory relationship, leading to the trainee needing to be placed in a different training location with a new supervisor [15]. In the absence of quality educational alliances, it has been suggested elsewhere in the literature that learners may not feel safe to disclose vulnerabilities, or to accept fully the feedback given from their supervisors [16].

It has been argued that it is the trainee’s, rather than the supervisor’s, appraisal of the quality of the alliance that is particularly important [17]. Their multifaceted judgement of the supervisor’s commitment to the alliance, throughout the course of the supervisory relationship, may impact the trainee’s engagement with teaching and learning in the context of supervision [17]. This suggests that the trainee plays an active role within the educational alliance and makes a case that, to best study supervisory relationships, the trainee’s contribution is important to consider alongside the supervisor’s role. This is a perspective that has frequently been overlooked in studies of the supervisory alliance [13].

This research therefore explores the lived experiences of United Kingdom (UK) postgraduate General Practice trainees as they engage in supervision within the complex clinical educational environment.

### Conceptual approach

Bordin’s working alliance based model of supervision was developed from the field of counselling psychology, but is frequently regarded as a ‘pivotal, transtheoretical construct’ in guiding the practice of supervision, regardless of its origin (13p20). It has become a model of ‘good’ supervision, where the alliance between trainee and supervisor is viewed as central to the health and quality of supervision, and provides a useful lens to explore areas of mismatch and tension. However, a singular focus on the micro-level interaction between trainee and supervisor may risk neglecting the influence of external structures, which can govern behaviour and experience [18].

Socio-cultural approaches in research frequently focus on how individuals shape their identity within the learning environment [19]. There is a growing acknowledgement that socio-cultural research can be profoundly informative when we attend to the conflict and tension that is present in learning communities, foregrounding matters of position, power and agency that may not be typically central in sociocultural research [19]. In the complex workplace-based educational environment of clinical medicine, which is situated in history, hierarchy, structures and processes, this may be particularly pertinent. Attempts to understand the supervisory alliance outside higher education settings are limited, and workplace considerations frequently neglected in study design [13]. With this in mind, we have taken a socio-cultural approach, applying a critical lens to consider notions of power, agency and position [20–22].

Positioning theory was used to consider the mutuality of the trainee’s development within the socio-cultural environment. Positions in this context are defined as:

“beliefs about the rights and duties of individuals in specific moments and social and material conditions” ([23] p1186)

It has been argued that the governing structure and culture offers these particular social positions, and calls on individuals to occupy that position [24]. Within a culture, we afford (or offer) different subject positions to one another by the way we talk, the activities we value and the outcomes we recognise ^(25p.26)^.

### Positioning

When considering positioning, we must consider the ways in which individuals are positioned (and position themselves) within a culture. Secondly, it is important to think about how such positioning comes about.

Bamberg offers a useful means to consider the ways in which individuals can position themselves [26, 27].

1. Level 1: Positioning the various characters within the socio-cultural context, in relation to one another.
2. Level 2: Positioning of the self (as the narrator) with respect to the audience in the interactive context (*e.g. the researcher as audience in an interview context*).
3. Level 3: The narrating of ‘self’, with respect to “Who am I”? This level relates to the way in which the narrator (individual) positions themselves to themselves, with regards to the predominant narratives.

### How does positioning come about?

One means by which positioning can occur is discursively, through talk and discourse. If can be argued that “*all elements of speech (alongside its content) constitute signs of the speakers claim to social position*” (25p.12). Put differently, the way in which we talk to one another depends upon negotiations and manoeuvrings related to the position we hold. We are therefore aiming to assert our position (and that of others) within talk.

A second means by which positioning can occur is through the notion of access. Such access, or inclusion, can relate to aspects such as space, activities, time and associates [25]. There is a sense that not everyone is afforded the same access depending on their particular social position.

This paper explores the following research questions:

1. How do supervisory relationships contribute to the positioning of postgraduate GP trainees within the socio-cultural context?
2. What is the trainee experience of power and agency in postgraduate GP training?

## Methods

In the UK, every GP trainee is allocated an educational supervisor, who is responsible for oversight of the trainee for the duration of their 3-year training programme. They host the trainee at their practice for the final year of training, providing clinical supervision [28]. In 2017–2019 a series of narrative interviews were undertaken with 13 GP trainees at the beginning and end of their final year of training. Pilot interviews were initially undertaken with 3 final year GP trainees, and participants were invited to tell their story of the contribution of supervision to their postgraduate training. We aimed to undertake two interviews during the final year of training (at least 8 months apart), to build rapport with participants and appreciate their life history alongside their stories of supervision, with an option for clarification in follow-up interview [29]. The interview schedule was designed to enable each participant to set the agenda for the stories that were told, avoiding disruption from the interviewer where possible [30, 31].

### Participant recruitment

Participants were sampled from one of the largest training regions in the UK, with an estimated 250–300 trainees in their final year of training [32, 33]. Participants were purposively sampled based on a range of performance outcomes on their end-of-year panel assessment (the Annual Review of Competency Progression, or ARCP), gender, training location and training status (full time, FT, or less than full time, LTFT), with the aim to capture a range of training and supervisory experiences, both positive and negative. Table 1: Interview Timeline (below) outlines the timeline of the initial and follow-up interviews.

**Table 1:** Interview timeline

Participant ID	Pseudonym	LTFT?	ARCP/programme performance (participant reported)	Interview 1 (stage of training)	Interview 2 (stage of training)
1 (pilot)	Raj	N	CSA fail	End of ST3	n/a
2 (pilot)	Jas	N	ARCP progress concern	End of ST3	n/a
3 (pilot)	Preet	N		End of ST3	n/a
10	Seema	Y	ARCP progress concern, CSA fail, AKT fail	Late ST2	Mid ST3
11	Sarah	Y		Mid-ST4 (after CSA)	n/a
12	Nat	N		Early ST3	Late ST3
13	Alison	Y		End of ST4	n/a
14	George	N		Early ST3	Post-CCT
15	Esther	Y		Early ST3	Late ST3
16	Stephen	N		Early ST3	Late ST3
17	Nadia	Y		End of ST3	n/a
18	Ayesha	N		Early ST3	DNA
19	Cara	N		Early ST3	Late ST3

*Abbreviations*: *N* No, *Y* Yes, *CSA* Clinical skills assessment (summative examination), *ARCP* Annual review of competency progression (annual assessment of progression towards qualification, considered by a panel), *AKT* Applied knowledge test (summative examination), *n/a*: Follow-up interview not applicable (trainee already at the end of their training), *LTFT* Less than full time, *Post-CCT*, After the completion of certificate of training (after qualification), *ST4* Some GP trainees have an additional year of training to pursue specific interests, such as research or leadership. This additional year is to enhance their breadth of experience, It is not related to an extension as part of remediation, *DNA* Did not attend for follow-up interview (and declined requests to reschedule)

All interviews were undertaken by a single researcher (DJ), audio-recorded and transcribed verbatim [34]. Field notes were made immediately after each interview. The interviews were typically around 30–40 min in duration (ranging from 14 to 72 min).

### Data analysis

We applied Brown and Gilligan’s Listening Guide to data analysis and adapted this analytic approach to incorporate a consideration of power, positioning and agency.

The Listening Guide involves 4 sequential readings of the narrative to attend to the different voices within it, and the relationships of the individual with the wider social world [35–37]. The first reading involves a consideration of the main ‘characters’ within the narrative, the broad themes and story being told and the researcher’s response [36]. The second reading concentrates on how the participant speaks about themselves. Attention is focused on the use of the pronoun ‘I’ within the narrative using ‘I Poems’. This technique involves drawing out ‘I’ statements as they occur chronologically within the narrative, and arranging them into stanzas [35]. Reading 3 attends to the key relationships in the narrative, and reading 4 to the influence of the wider socio-cultural context [26, 27].

Bamberg’s 3 levels of positioning were applied to separate readings of the Listening Guide. These explore positioning between the various characters in the socio-cultural context (Level 1), between the narrator and their audience (Level 2), and the narrating of ‘self’ with respect to ‘who am I? (Level 3) [26, 27].

Table 2 (Applying the Listening Guide to Our Research) outlines our approach to the analysis, which was applied to each narrative individually, and then across the narratives

**Table 2:** Applying the listening guide to our research

**STAGE OF READING (‘LISTENING’) **	**CONSIDERATIONS AT EACH STAGE OF READING** (*adaptations to consider positioning, power and agency)
**1 – The Plot**	Focus on the broad story and themes What stories are told? Who is present? (Who is not present)? What is my response (as the researcher)? ^a^*Positioning: to me (as the researcher) (Bamberg’s Level 2)*
**2- Listening for the ‘I’**	I-poems Draw out ‘I’ statements (short phrases containing ‘I’) as they occur chronologically within the transcript Arrange these into stanzas ^a^*Positioning: to themselves (Bamberg’s Level 3)*
**Additional step – Contrapuntal voices**	Consideration of the tones and musicality of the narrator’s voice Consideration of how these illuminate the broader story
**3-Relationships**	What are the key relationships within the narrative? How are these viewed? How is the individual influenced by these relationships? ^a^*Positioning: to their supervisor and the wider training practice team (Bamberg’s Level 1)* ^a^*How does this relate to access and agency?*
**4- Structural and Cultural context**	What are the wider system ‘voices’? How have these influenced the individual? ^a^*Positioning and vantage point within the wider system* ^a^*How does this relate to agency, access and artefacts?*

^a^Adaption of Listening Guide, drawing on concepts of positioning

### Looking across the narratives

Key to our analytic approach was the appreciation of each participant’s lived experience, situated in the socio-cultural context. However, we also wished to look specifically at stories of conflict or tensions with supervisors, and to explore positioning, structure and agency within these stories. Using the modified listening guide as a framework, an approach was undertaken to ‘listen’ across the narratives. Overarching stories (identified in the first reading of each narrative) were outlined. Each was labelled and summarised using information gleaned from readings 1–4 of the narrative, such as characters involved, participant voice used, aspects of agency or key relationships in the narrative. Using these story summaries, it was possible to compare and contrast trainee experiences across the narratives.

### Ethical considerations and reflexivity

The project was approved by the University of Birmingham Ethics Committee ERN_14-0957A. Reflections on personal elements of reflexivity, the methodological approach and the way in which the discipline of interest (GP supervision) has formed and developed were considered [38, 39]. Regular team discussion (DJ, JB and ID) and inclusion of the researcher’s voice in the first reading of the Listening Guide also contributed to reflexivity [38, 40].

## Results

The results outline key elements of positioning theory within our data, such as the positions held by participants, the supervisor and training environment contribution to the positioning of trainees, and the role of power and agency. The authors have incorporated their interpretations of the narrative accounts, alongside participant quotes (verbatim), to illustrate the multiple voices attended to within data analysis.

### Positioning

The trainees occupied variable positions within their training context, and some of the trainees assigned these labels to themselves.

Cara referred to herself as an ‘unpaid salaried doctor’ when recounting difficulties related to heavy workload. She felt her practice relieved heavily on trainees to manage patient care, utilising them as already-qualified GP’s (‘salaried GP’s’), instead of as training GP’s:

‘It’s just they treat you like an unpaid salaried doctor. And that’s why nobody wants to do salaried work, cos they see what they can treat you like’ (Cara)

Seema referred to herself as a ‘problem trainee’ in her practice, and felt that others (supervisors and other GPs at the practice) saw her this way also:

‘Because  I was a problem trainee here. So people knew that I was a problem trainee’. (Seema)

George’s interview suggested ‘insider’ positioning, and a ‘near-peer’, social relationship with his supervisor and colleagues, where his relationship with his supervisor grew to one that he viewed as a ‘friendship’:

‘it’s really nice having come out of the other end to still be in touch with my supervisor from last year and my supervisor from the year before actually. And seeing them socially recently. And that’s really nice. That’s one big thing about the recent supervision I’ve had. It’s kind of you know, it builds…

it does build friendships that last which is nice’ (George)

In contrast, Seema’s narrative suggested an ‘outsider’ position, where she frequently felt lost or confused about what was expected of her. She struggled to build relationships with her supervisor or colleagues, and found them to be inaccessible for support or questions. She recounted a story where she felt frustrated that her supervisor judged her for not progressing quickly enough in her consultation efficiency, towards the standard 10–15 min timeframe. She reflected that some of her delays were caused by struggling to access support from her supervisor when she needed it, and recalled the need to stand outside her supervisor’s consulting room door for a long time, waiting for them to finish with their patient before they would provide clinical support for her questions:

“they said, “You’re still on half an hour [appointment duration] and you haven’t done anything about it…”.

And I said, “Ok, about [the] half an hour, I’ll try and do [action].

But I must admit to it, ***the waiting outside your room [to get help or advice]*** sometimes takes 10 min…”.

So, they said, “No…. I see patients very quickly”

I said, “You might…***but I still have to wait outside***” (Seema)

### Supervisor contribution to positioning

Supervisors contributed to the positioning of trainees in different ways. Most critically, positioning occurred through the degree of access afforded to the trainee, particularly in terms of the time, resources and associations with supervisors and members of the team. George’s experience as a ‘near-peer’ (referred to in his quote above) illustrates a relationship with his supervisor where he was invited out socially and remained in contact with his supervisor even after qualification.

In contrast, Seema’s reflections (outlined in her quote above) depict an instance where access to her supervisor’s support was limited, and where her ‘outsider’ position was emphasized through standing outside the door. The I-Poem below, taken from a later excerpt from her interview, highlights a paradox Seema faced. Her supervisor required that she came to discuss each patient with him, but she found him to be frequently inaccessible. In one instance, she reported that he’d ran away from her, shutting his door before she could ask him a question:

**I-Poem – ‘I saw him, he ran’**

I was supervised.

I was supervised

Every patient I see

I have to discuss that.

So every time I used to see a patient

I used to go

I was taking my half an hour

I’m thinking

I saw him, he ran.

I have to call

And I couldn’t get the look

I came back, went back, came back, went back

Another method of positioning was through discourse; in conversations with supervisors and staff, and also by the way in which the trainees were referred to by educators (sometimes in written communication). Alison recounted an experience where she had felt overworked by her practice, and had sought help from her supervisor for what she felt to be an unfair and unrealistic workload. Her supervisor had written in her educational portfolio that she was ‘not coping’, a label she disagreed with:

*…he put like an educators note on my portfolio that I felt was very um, unfair. Didn’t really represent the true situation…in the educators note for example he wrote um, “we’ve agreed to reduce her admin load”. ****So it just sounded like, “she’s not coping…”.***

*…****Which I felt was quite an unfair representation of the situation****. So all in all I felt quite disillusioned with his supervision and practice. I feel like I’m not really one of those people who crumbles at the smallest thing. Um, I’m not someone who is constantly asking for dispensations. I usually just get on and do the work… (Alison)*

Esther described an experience where her supervisor had a positive influence in positioning her as an ‘insider’ within the practice, guiding her relationships with others through introductions:

*Even right from the beginning, every time I went, ****he’d walk me round the building and introduce me to everyone****. So by the time I was going back after maternity leave, I really felt like I knew the place. Even though I’d technically never worked there…*

*…but I felt like I already knew it, and knew everyone there, because of how he’d kept introducing me to everybody.*

*(Esther).*

*Influences of the wider training system*

Some of the GP trainees appeared to lack clarity, or feel suspicious, about the supervisor’s goals and agenda in supervision. In these instances, supervisors were perceived as agents of the wider ‘system’, rather than as supporters of the trainee. Jas outlined an experience with her supervisor where she experienced an examination failure, but doubted the authenticity of her supervisor’s support:

‘So, when I failed that ARCP,I got lots of… messages on my portfolio and recorded, documented, evidenced-based things on my portfolio from my supervisor like 2 weeks before…

***And I felt that was just to show***. Just to show that actually “I have tried to kick her up the backside, but it’s not working”. But just 2 or 3 weeks before. Which I thought was a bit nasty. ***Because I’d not really been approached on a personal level***’. (Jas)

The wider members of the training practice team also appeared to have a prominent influence on supervision, and this influence often required navigation and negotiation. Some of the narratives illustrated the potential for the training practice to constrain resources, scheduling or opportunities for supervision, such as in Nat’s experience:

‘Yeah. Not having scheduled tutorials frustrated both of us I think…

…Because they [training practice] wouldn’t allocate him [supervisor] time in the rota for it, because we hadn’t given 6 weeks’ notice. And I think it’s the lack of understanding between HR and admin and the trainers as to what needs to happen. …

.. No matter how many things he’s tried and sent emails and spoke to people. Certain things just haven’t happened or have been far too difficult. (Nat)

Others, like Nadia, had contrastingly positive experiences, where education and supervision were prioritised and facilitated within the practice culture:

‘…if I have problems I can talk about cases”. ***And we have the opportunity***, because we have 2 practice meetings on a Monday and a Friday, and we can discuss cases then. And I’ve always found that useful ***because everybody is there.***The salaried GP’s, the other trainees, um the nurses, the partners…so it’s quite useful to be able to just bring them. And they do encourage us to talk about cases. And even the partners will bring the cases that they’re not sure about. ***So, you do kind of feel like everybody’s on an equal or level playing field’. ***(Nadia)

### *Power and raising concerns*:

Some of the trainees chose to raise concerns about quality of their training and supervision, escalating these through formal complaint channels. Some, like Alison, felt that escalation had led to little change in their circumstances:

[Alison made a complaint about her supervisor to her programme director]:

‘but then, all his suggestions never appeared on paper and ***became muted ***by the time that they were brought back to the practice. So actually the things that he thought were unfair about how the practice was pushing me along. He said to me, and that felt very supportive, but then ***he never told the practice that or told my supervisor that. And I think that I needed that advocate. ***(Alison)

Alison was later deterred from escalating her concerns further due to worries she would be perceived as causing ‘trouble’:

‘I said I’d only probably want to do it once I’ve finished training. ***Cos I don’t really want to get myself into trouble’. ***(Alison)

This sentiment was also shared by Cara:

‘***I don’t think it helps you ***to be moany’ (Cara)

Raj raised concerns about a member of staff at his training practice through formal channels. Although this led to action and a change in placement, he had mixed feelings about the escalation, feeling ‘bad’ about the new situation:

So I’ve ended up coming to a new practice in ST3, um, with a new supervisor um, who um, hasn’t known me for very long. Um, ***but I appreciate it’s a difficult situation for both him and myself ***and my previous practice. So we’re all just trying to work through it together really so…

… I mean my supervision experience here has been short. But, you know, the educators here are always approachable, they ‘re really friendly, um, they did what they had to do within the three weeks to get me through my educational supervisor review. So,you know, I can’t fault them here either. ***I just feel, part of me feels bad for landing them in kind of a difficult situation***. (Raj)

### Trainee agency

Instead of raising formal complaints, some trainees used other means to navigate difficulties in training and supervision. The electronic training portfolio (eportfolio) was one method used to ‘defend’ through documentation, by collating evidence and recording concerns. Upon reading her supervisor’s note on her portfolio describing her as ‘not coping’, Alison chose to respond using the same EPortfolio platform:

… ‘Yeah, so when I’d gone to my supervisor with all my problems, and he hadn’t been particularly helpful, I then, so ***I wrote a comment on my portfolio ***as well. (Alison)

The surgery timetable also served as a means for trainees to navigate problems they perceived with workload and supervision. This is an electronic appointment booking system, outlining the daily workload for a trainee. Mastery of the surgery timetable was an important turning point in Cara’s experience of moving away from being overworked within her practice, through learning to document and allocate time to unscheduled work.

‘I think at the beginning, when people would come and knock on my door, which they do 5, 6 times a clinic, I was being snappy with nice people.

“Can you come and review this wound? I‘ve just got this prescription. We’ve got a phone call”.

And I’d be like “agghhhhh” [frustrated sound].

Whereas now I’ll just say, “Yeah, I’ll review the wound”. ***And I will block a slot***and I will see that patient and do it properly…

I’ve got a bit of more backbone now. To say, “Yes, I can be helpful, but ***I have to protect myself as well***”. (Cara)

Notably, the consulting room door was an important tool in offering or denying access to supervision in Seema’s story. In a new placement, she reflected on how she’d learned to access support from her supervisor in new ways; no longer relying on knocking on their door, but rather mastering other means of accessing support.

‘So they are supporting me…

Every patient here also was discussed, but ***I could send a text ***that I’ve seen the patient, can I come over***. If they’re not free, I can sit in my room***; finish my documentation, rather than waiting outside of their door and coming back and doing, so of all things that has changed’. (Seema)

### Changing perspectives

A number of the trainees (Preet, Nat and George) reflected on how their supervisory relationships had improved over time, as they got to know their supervisor better (and were known better themselves). Preet reflected that part of this involved changing her expectations about supervision, and engaging in negotiations with her supervisor to find a ‘happy medium’.

“It got better in the later half of the year as I said. My relationship isn’t as bad as it sounds! (laughs). We actually do get on, em, and ***we found a nice happy medium***. I think it was just a bit of ***a personality and expectation clash***. Em (2), yeah. So supervision wasn’t bad as such. Em, it just wasn’t as helpful as it probably could have been. (Preet)

Trainee perspective also appeared to change through moving to a new placement, with a new supervisor. In Seema’s first interview, she struggled to appreciate what her supervisor wanted from her, and frequently spoke in what we labelled as a ‘defensive’ voice, seeing herself as a victim in the interaction. This is outlined in the I-Poem below:

**I-Poem: defensive voice**

I sat down to discuss it.

I said, because it was you who put the note on

I don’t care who said it.

I’m not taking it offensively.

I’ve been asked to discuss it

I want to improve myself

I will never know what to improve until you tell me

I can challenge you

if I’m late by more than 5 min

I can challenge

I can

I’ll do whatever

I never got the explanation

The I-Poem below (taken from her follow-up interview, when placed with a new supervisor) outlines a change in her vantage point, where she was able to see things from her supervisor’s point of view. Although the new supervisor also required her to discuss each patient with them, she appreciated why this was needed:

I poem- a different perspective

I can’t complain

I can understand

I could send a text

I can sit in my room

If I was a GP supervisor

I would do that

I wouldn’t take anything in my hands

If I don’t know the trainee

I wouldn’t

I would see

I can’t complain

## Discussion

Our results suggest that supervisors have a prominent role in the positioning of trainees, with the power to deny access to support or resources for training, and to label trainees to other stakeholders through either verbal or written communication. Some of the trainees, like Esther and George, felt they benefitted considerably from arrangements where their supervisor could help them to broker ‘insider’ relationships with the wider practice team, or where they provided greater access to themselves through a near-peer relationship of role-modelling and support. However, other trainees felt frustrated by the positions they found themselves in. Seema frequently struggled to access support from her first supervisor and was seemingly positioned as an ‘outsider’ and a ‘problem trainee’. Alison and Cara described their own labels of ‘not coping’, and an ‘unpaid salaried GP’ within their training practices, which they did not agree with.

For some of the participants, like Jas, supervisors were perceived as agents of the wider institution, rather than as advocates for the trainee, leading to suspicion, feeling ‘unsafe’ and disengagement with proffered supervisory support. Trainees who did attempt to overtly challenge these positions, through complaint or discussion with their supervisors, often perceived the outcome to be unsatisfactory, and at a cost to themselves. Trainee agency to work out their position in the context of supervision appeared most evident for those who mastered particular artefacts of training, such as the electronic appointment book and the training portfolio, rather than those who challenged situations directly through formal processes.

### Theoretical implications

One means by which positioning can occur is discursively, where “*all elements of speech (alongside its content) constitute signs of the speakers claim to social position*” (25p.12). Although there was some evidence of positioning through verbal discourse in our results, we more frequently observed the use of written communication (through the electronic portfolio) to negotiate and manoeuvre positions. This provides a useful insight to the socio-cultural context in GP training, where both trainee and supervisor appear to place great emphasis on documentation and evidence. In our earlier research with GP supervisors and a review of the UK GP training documentation, a heavy reliance on written communication was also demonstrated, typically as a means to protect supervisors from potential complaint, and to satisfy institutional requirements on a number of measurable metrics [15, 39].

A second means by which positioning can occur is through the notion of access. Such access, or inclusion, can relate to aspects such as space, activities, time and associates [25]. We observed that the trainees had variable experiences of access to supervisor and team support at their training practices, despite sharing the same job title and training grade. Some supervisors made concerted efforts to broker the trainee into the practice community, through introductions and invitation. The structures and processes within the training practice itself also contributed to access, through their role in timetabling, location of trainees, arrangement of team meetings and appointment book management.

Whilst individuals are susceptible to situational determinants of the culture (and thus the social positions afforded to them), they *can* afford themselves self-control and agency if their sense of identity is challenged [25]. Proponents of heuristic development refer to this as ‘improvisation’; a process whereby the individual can respond to the situation in creative and imaginative ways [25]. Mastery of the artefacts of training, such as the electronic appointment book, portfolio and access to the supervisor (that didn’t rely on knocking their door) were ways used by trainees in our study to improvise and respond to challenging supervisory situations, which appeared more fruitful than overt challenge or complaint.

Key to the concept of positioning is that the various participants will occupy different positions in the social world, and therefore hold different perspectives on it [25]. It has been argued that the individual can only look at the world from the vantage point that they have been afforded, and thus from the social position from which they are persistently cast [25]. This perspective is not fixed and develops over time. Seema’s early narratives of an ‘outsider’, struggling to access her supervisor and understand their requirements contrast with her later reflections and changed vantage point. In the new placement, she was able to contact her supervisor in a variety of ways for help and was able to appreciate the need for her new supervisor to oversee and monitor her performance.

### Implications for supervision

**Fig. 1:**
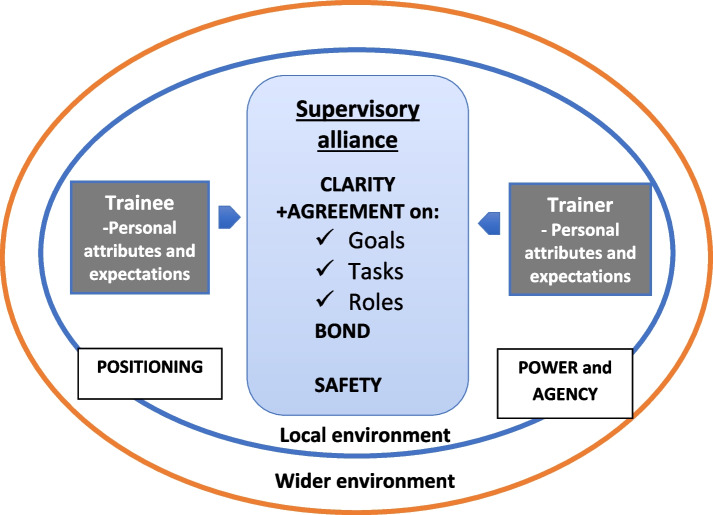
Model of the supervisory alliance in postgraduate GP training (MSA-GP): a springboard for discussion

Bordin’s working alliance-based model of supervision outlines the importance of dialogue between trainees and supervisors; to negotiate and agree on the goals and tasks of supervision, in the context of an emotional bond [12]. However, our results illuminate additional nuance and complexity with respect to power relationships, positioning and wider practice and institutional influences on postgraduate GP supervision. Figure 1 (Model of the Supervisory Alliance in Postgraduate GP Training (MSA-GP): A springboard for discussion) draws on our findings to develop Bordin’s model.

#### Local and wider environment

For educators who wish to develop strategies to support quality supervisory alliances, we would caution against a focus on the trainee-supervisor interaction without consideration of the socio-cultural context. Supervision, in the context of our research, did not operate in isolation. Supervisory relationships appeared constrained or facilitated by wider professional expectations and by the practice team, which also had a role in the positioning and support of trainees. Supervisors appeared (at times) to be perceived as linked closely to the wider training ‘system’, and were perceived by some trainees as agents of structure. The influence of documentation has been described elsewhere in the literature as a threat to primary care supervision, in relation to workload and unwieldly software [41]. However, our results have also suggested that the emphasis on collation of evidence by professional education bodies may further threaten the supervisory interaction, used instead of discourse as a medium to communicate or defend.

#### Power and positioning

Bordin’s working alliance-based model of supervision outlines the importance of dialogue between trainees and supervisors; to negotiate and agree on the goals and tasks of supervision. The supervisor’s contribution to positioning of trainees in our research, and the difficulties experienced by trainees when raising formal concerns about their supervisors, indicate that power relationships also need to be acknowledged and explored in supervisory conversations. Power imbalance has been considered to be a potential threat to supervisors and trainees in reaching agreement on the goals and tasks of supervision [42–46], and the assessment and monitoring role of the supervisor has been suggested to exaggerate this imbalance [4, 6, 42, 44]. Our results also indicated instances of trainee suspicion around the supervisor’s commitment to their development, perceiving supervisors to be agents of the wider structural institution, further inflaming perceptions of power imbalance.

#### Safety

Supervisors frequently rely on trainee openness to undertake needs analysis and to tailor support [47], yet only a few of the trainees in our study had raised their concerns directly with their supervisor or training body. Non-hierarchical relationships are advocated to minimise power imbalance in GP supervision [42, 48, 49], and can be fostered through trainee feedback to supervisors [43, 50]. Reassurance from supervisors has been viewed to create safety within the supervisory relationship, encouraging trainee openness [51].

#### Clarity

We also observed that some of the trainees appeared to lack clarity regarding their supervisor’s expectations. For example, Seema described a paradoxical situation where she was obligated to discuss every patient with her supervisor, but found it difficult to understand how she could access him for that level of support. On moving to her new practice, she was given greater clarity on gaining access to her supervisor, such as sending a text message, which helped her to navigate this challenge. Elsewhere in the literature, supervisors have historically found it difficult to articulate their teaching approach, resulting in trainees lacking clarity [43, 52–55]. We would recommend that supervisors are explicit about the trainee’s purpose in the training workplace, and about what they are trying to achieve in supervision [52, 56]. This should also include clarification on their multiple roles (including their assessment role) [44, 57], and how the trainee should access help [5, 42].

### Agency and artefacts

The positive stories of supervision in our research indicate that supervision also has significant potential to facilitate and develop trainee agency, through the affordances of access, creation of safe spaces to share vulnerability and through brokering inclusion in the wider community of practice. This mirrors findings elsewhere in the literature, which refer to the role of the supervisor as one that must broker the relationship with the trainee and practice community, whilst overseeing patient care and creating safe relationships to facilitate trainee disclosure of weaknesses [3]. Artefacts of postgraduate GP training in our study, such as the electronic portfolio and appointment book, may offer additional windows to areas where supervisors can offer significant clarity and support for trainees in their orientation and development.

Supervisory discussions within UK postgraduate GP training are typically framed to explore the trainee’s learning needs, reflect on their performance and develop action plans for development [58, 59]. However, probing beneath the surface to consider the expectations and experiences that influence professional behaviours or values is not typically articulated within training guidance. We have developed the model of the supervisory alliance in postgraduate GP training (MSA-GP) (Fig. 1) to help facilitate meaningful conversations for trainees and supervisors.

### Limitations

The aim of the narrative interviews was to provide a rich account of the complexities of supervision and its contribution to trainee development, across a range of trainee experiences. We did not seek to provide generalisable findings, or to make claims about the representativeness of the trainees and the stories they told [36, 60, 61]. At the time of the interviews, the lead researcher (DJ) was a newly qualified GP. Reflection on this position as a researcher required a consideration of the access and position afforded to the researcher by the research participants, and also of how participants positioned themselves within the narratives [62]. The voice centred approach offered a means to consider this within data analysis, and regular research team discussion also contributed to reflexivity [26, 27].

## Conclusions

Supervisory relationships appear to remain an important contribution to trainee development, but are influenced themselves by institutional expectations, inherent tensions and the socio-cultural context.

This paper offers a model (Fig. 1) to serve as a springboard for discussion for trainees and their supervisors. The model develops Bordin’s working alliance based model of supervision, where quality supervisory relationships were viewed as encompassing agreement on the goals and tasks of supervision, in the context of an emotional bond [12]. Currently, supervisory discussions within GP training are supported by an educational supervisors’ review framework which appears to align with Bordin’s model. The framework supports supervisors to discuss their trainee’s learning needs with them, reflect on their performance and, together, they develop action plans on the goals and tasks for trainee development [58, 59]. However, discussions that focus only on these areas may fail to sufficiently address underpinning assumptions or expectations for quality supervision, and could lead to a lack of clarity, safety or openness for the trainee.

Our model of the Supervisory Alliance in Postgraduate GP Training (MSA-GP) is designed to encourage supervisors to think more broadly in their relationships and discussions with trainees. We recognise that both trainee and supervisor will enter supervision with particular attributes and expectations, which may align or differ, and which may not be always obvious or shared. At the interpersonal level (the centre of the model) we illustrate the importance of nurturing clarity on the expectations that the trainee and their supervisor may have about supervision, and the roles of supervisor and trainee within supervision. This might involve a discussion around the responsibilities and roles that each party holds outside of the training relationship that may have a bearing on supervision, or sharing expectations about who should be responsible for setting the agenda or learning content of meetings. We also encourage supervisors to consider how they can foster psychological safety to encourage openness in supervisory discussions, reflecting on how they may facilitate a non-hierarchical relationship, taking steps to reassure their trainees on performance and inviting their feedback. The model also highlights the need for supervisors to acknowledge and discuss the contribution of the socio-cultural context (illustrated by the concentric circles in the model), and the role of power, agency and positioning within this. This might involve brokering relationships between the trainee and members of the wider training practice, a review of the timetabling of supervisory and patient-related activity, or helping the trainee to master the artefacts of training (such as empowering the trainee to use the appointment book to enhance their learning opportunities). The goal is to establish a platform for greater clarity and negotiation of the inherent complexities within postgraduate GP training, and to assist in making explicit the taken-for-granted assumptions about training and supervision.

### Supplementary Information

**Additional file 1:Appendix 1.** Interview schedule for narrative interviews with GP trainees. **Appendix 2.** Story summaries. **Appendix 3.** Exemplar of narrative analysis summary.

## Data Availability

The datasets generated and/or analysed during the current study are not publicly available due to the risk that individual privacy may be compromised but are available from the corresponding author on reasonable request. Data supporting the results of this study have been provided in Additional file 1: Appendices 2 and 3.

## Abbreviations

AKT: Applied knowledge test
ARCP: Annual review of competency progression
CCT: Certification of completion of training
CSA: Clinical skills assessment
DNA: Did not attend
GP: General practitioner
LTFT: Less than full time
MSA-GP: Model of the supervisory alliance in postgraduate GP training
ST3: Speciality trainee, year 3
UK: United Kingdom
