# Supplementary material for: Positioning, power and agency in postgraduate primary care supervision: a study of trainee narratives
Source: BMC Med Educ. 2023 Nov 17;23:880. doi: 10.1186/s12909-023-04826-9 (PMC10656937; doi:10.1186/s12909-023-04826-9)
Supplement: Supplementary file 1 — Additional file 1:Appendix 1. Interview schedule for narrative interviews with GP trainees. Appendix 2. Story summaries. Appendix 3. Exemplar of narrative analysis summary. [file 12909_2023_4826_MOESM1_ESM.docx]

# Appendix 1: Interview schedule for Narrative Interviews with GP Trainees

INTERVIEW SCHEDULE – summarizing the series of interviews

***What’s Happening in the GP Trainee-Trainer Supervisory Relationship?***

***Beginning:***

***Short questions to determine where participant is in their training:***

Just to clarify before we begin – where are you up to in your training? (i.e. regular run-through. FT/LTFT? Extension/out of sync).

***Introduction***

Thank you for agreeing to take part in this interview.

The study aims to explore the GP trainee-trainer relationship, and how it addresses the training needs of GP trainees, particularly in their ST 3 year.

You’ve been asked to take part because you are a GP trainee, who therefore has experience of the GP trainee-trainer relationship, and the training needs of GP trainees.

*If regular training:*

The series of interviews hopes to explore your experiences. (It’s aimed that I’ll talk to you today, and again after you’ve sat your CSA). It may be that I contact you a final time to check things with you, or ask for some clarification if that’s needed.

*OR (if towards the end of their training)*

In the initial invitation, I’d discussed the plan to have a second interview after CSA. In your case, this isn’t relevant, as you’ve already sat your CSA and are coming towards the end of training. As a result, the interview today will be the only one that we will do.

Each (the) interview should last around 45minutes.

*Opportunity to ask questions*

*Opportunity to review Participant Information Sheet*

*Signing of consent*

**INTERVIEW ONE**

Today’s interview is to focus on your experiences of medicine and training before becoming an ST3 GP trainee.

It involves some looking back to the past, so take time to think where you need it. I’ll give you a pen and paper too, as I know some people think better with a pen in their hand!

This type of interviewing is very different to the way we take histories in General Practice, so please do talk freely. It is also different to a typical interview, as I don’t have a predefined list of questions to ask. I am much more interested in hearing your story and the things that you think are important.

You may notice me scribbling here and there – that’s just to jog my memory in case I need to come back to something you’ve said later.

OPENING QUESTION

- Please can you tell me the story of your supervision experience from medical school until now? Start however you like, and consider the experiences, events and people that have shaped that story, and which you think are important to share.

OR *(if coming to the end of ST3):*

- Option to start from ST1 if too difficult to go back in terms of time.

*It is likely that much of the interview will be based on the given response to this question.*

***Should the respondent require additional prompting, the following may be considered:***

*What happened then?*

*Is there anything else that you think is important to tell me about your training? (Encourage expansion of the narrative).*

**If more prompting needed:**

Consider: are there particularly important events in your training experience?

Are there particularly important people in your training experience?

*If struggling to construct in entirety, consider:*

Story of medical school

Foundation years

Becoming a GP trainee

GP training so far

The context: family, culture, past experience, the “personal”, institution/external factors

Exploration of expectations of supervision

Exploration of supervision experiences in training –good, bad, best, worst

Agreement in supervision

Roles in the relationship

Working alliance

Relation to performance

## Appendix 2: Story Summaries

# Appendix 3: Exemplar of Narrative Analysis Summary

Seema: Analysis Summary (researcher reflections in red)

**Seema: Participant 10 Summary** 72 mins

**Story Caption: I’m a problem trainee, what’s wrong with me?**

**Brief Synopsis**

The participant in this narrative is an older trainee, single mother and an International Graduate. She has spent a number of years training within a hospital speciality, but changes to General Practice training later in her career.

Her training journey appears fraught with difficulties (including hospital posts outside of General Practice). These include supervisors raising concerns related to her timekeeping, rota commitments and competence. Later, in GP training, she has a breakdown in relationship with her trainer, fails her AKT and is referred for Professional Support.

**Main stories (First reading)**

| **The courtroom: a victim** | The trainee describes a training journey fraught with difficulties. In this narrative, she is defending her position as the victim, with the training culture as the protagonist (and various supervisors seen as agents of the training culture). |
| --- | --- |
| **What’s wrong with me?** | The quieter, vulnerable voice: In this narrative, the trainee appears to be questioning why these difficulties have occurred, and is asking herself, ‘What is wrong with me’? The narrative appears to be a place where she is working out her sense of self within a culture which has positioned her as an outsider. |
| **Seeking acceptance** | Trainee tries to adapt to behave ‘appropriately’ for her supervisor |
| **A new vantage point** | In her new practice, she is learning how to work in the identity of a ‘problem-trainee’ and move forwards, with support |

**WHO is telling the stories? (First reading)**

The trainee describes herself as a ‘problem trainee’. Certainly, her training journey has not been smooth.

There appears to be a catalogue of misunderstandings: smaller stories woven throughout the narrative.

**Positioning to me (as the researcher)**

Protagonist. In her eyes, supervisors have misinterpreted her intentions, and she has struggled to articulate a response.

It appears that she views me (the researcher) as a potential advocate, and someone who can listen to her ‘side of the story’. Her ‘side’ relates to her identity as ‘not a typical trainee’. Within this sense of ‘self’, she has the responsibility of motherhood (and being a single mother), which she must balance with the demands and expectations of GP training.

***I-poem “I am a single mother”***

*I started GP training*

*I was moving from Coventry*

*I was living in Coventry*

*I made him sit the exam*

*First choice was Birmingham*

*Which I never got*

*I had a few issues*

*I couldn’t be there bang on time at 8 o’clock*

*I have to drop my kids*

*I made it very clear*

*I moved into hospital*

*I never needed to go at 8*

*I went there at half 8*

*I was always bang on half 8*

***I-poem “I’m not a typical trainee”***

*I’m less than full-time*

*I’m part time*

*I’m quite an experienced doctor*

*I’m not training at the level of the trainees that come out just out of medical school*

*I’ve done *surgery (speciality changed to preserve anonymity)*

*I know what the training in the hospital is like in *surgery*

*I training in India*

*I’m very experienced*

**Vantage point:**

Outsider: seeking acceptance and inclusion

She perceives the institution and training culture as the antagonist, and many of her supervisors as agents of this.

Through her experiences, there is a sense she has come to expect judgement and scrutiny from the prevailing training culture and its agents.

She perceives the institution as having particular expectations of her (as a ‘good trainee’): Humble, teachable, engaged, and motivated

‘Insider’: The narrative ends with the trainee at a new practice, with a new supervisor. She explicitly contrasts her current experience:

*I’m still doing the same things. I haven’t changed anything since I’ve come here. Um, but I feel better. I feel better. I feel supported. I don’t know if it’s from the deanery. They have said you have to be supportive. Sometimes when I’m feeling under-confident. Or when I’m feeling like, “ok, no, I’m not a great person”, maybe that makes me think maybe it’s not me. Maybe it’s the deanery said that to them, that she has to go gently.*

Here, she describes her tentative journey to a different position – taking up more of an ‘insider’ position, but battling with her sense of self related to her previous experiences.

**Me as the listener:**

As the listener, I frequently find myself reacting to the defensive voice within the narrative. Not only is she attempting to defend herself to me, but she also recounts examples where she has defended herself to supervisors. As the listener, such defensiveness is jarring, and filled with a sense of frustration and almost aggression from the trainee. It creates feelings of empathy in me towards the supervisors involved. I wonder if, related to this defensiveness, she may well have ‘blind spots’ within her learning and professional development, and thus efforts of the part of supervisors to ‘teach’ or ‘support’ may not have been well-received. The defensiveness displayed, exam failure and instances of lateness or perceived lack of commitment, create in my own mind a picture of a ‘problem trainee’.

However, when I attend to the quieter, vulnerable voice, I feel a deep sense of sympathy for the trainee. From the outset, her identity of ‘not a typical trainee’ appears to have positioned her as an outsider, and seems to continue to position her in this way throughout the narrative. I find myself rooting for her to attain the acceptance and validation she craves within the community of practice and the training culture, and thus feel a deep sadness on the occasions where she finds herself rejected again.

**Expanding on the narratives (First and Second Readings, including relevant I Poems)**

**Narrative 1 – The courtroom. A victim.**

Supervisor as the antagonist

The starkest example of a supervisor as an antagonist within the narrative is her ST2 GP placement supervisor. The following excerpt is an example of this:

*“And then this meeting that happened it was very frustrating I would say, because somebody telling you that you don’t have a clinical knowledge, um, because you telling me to read everything. It’s very difficult to read everything. You are a GP, and I thought in my head, there’s nothing, there is no way you know 100% everything. Such a wide, you tell me what are the areas I should be focusing more than others. I can’t overnight in 6 months cover everything, If I’m not being very well educated.*

*So he said, first he fumbled.*

*Then I said, “No. You need to give me an example of a patient which I’ve done wrong. Because the way the meeting is happening, it’s really looking like I’ve done something wrong, so I need to know which patient has been neglected, mishandled”.*

Within this excerpt, the trainee’s ‘case’ is built and a defensive voice is paramount; rejecting the assumption of the trainer that she is a ‘bad’ trainee, and wanting to be understood as a ‘good trainee’. It is interesting that much of her ‘defence’ occurs ‘in her head’, suggesting that her voice is somewhat silenced in her interaction with her trainer. If the discussion did in fact play out as it is described, the supervisor would have simply heard her rejection of his diagnosis and her defensive call for ‘proof’. However, her search for more specific feedback from the supervisor is not expressed to him directly.

**Narrative 2 – What’s wrong with me?**

However, listening to the voice within the I-poems in the subsequent dialogue following this episode, a different perspective is offered, where the trainee’s vulnerable voice is apparent.

**I-poems – vulnerable voice**

*I’ll sort that out*

*I swapped it*

*Now I know that*

*I said*

*I didn’t think of that*

*I shouldn’t have*

*I said*

*I didn’t know that*

*I’ve discussed that*

*I didn’t know*

*I had opted*

This quieter voice provides a window to the second story within the narrative; a vulnerable voice. It appears that within the narrative, she is attempting to work out her identity and position, and is asking the question (of herself), “What is wrong with me”? The story here is a questioning of her sense of ‘self’ within postgraduate GP training, and in relation to her ‘bad’ experiences within training. It appears that, through the difficult experiences within training, she feels pushed into a position which is not aligned to the sense of self she desires. She refers to herself as a ‘problem trainee’, and positioned as an outsider within the Figured World of GP training.

**Narrative 3 – Search for acceptance**

Searching for acceptance –trainee agency

As the listener, the defensive voice is loud in this narrative, and the trainees describes occasions where this ‘defensive voice’ has been used to challenge her supervisor. As the listener, the sense of protest and defence is louder that I would expect, and I find myself quite jarred by the intensity and volume of the protest. I could also imagine that a supervisor may well feel the same – and conclude that their feedback and guidance is falling on ‘deaf’ ears.

**Positioning**

However, attending to the issue of positioning within the narrative, alternative perspectives are important.

The trainer is intending to direct and to guide, expecting humility and engagement from the trainee. However, the trainee expects acceptance and validation from the trainer, and perceives the trainer’s ‘guidance’ as judgement and rejection (thus positioning her as an ‘outsider’). Her response, often expressed defensively, is an attempt to gain the acceptance she craves (by rejecting the trainer’s claims of ‘bad’ trainee, and defending her desired position of ‘good’ trainee). Perhaps the trainee must defend so ‘loudly’ on the few occasions she describes, because of the degree by which she is positioned as an outsider, and frequency by which she is silenced within this position.

Sadly, as the listener, I wonder if this defensiveness may reinforce her outsider position, due to the way in which it is received by the trainer.

Search for acceptance- the olive branch

At a turning point in the story, it seems that the trainee begins to realise this. She describes an attempt to display the attributes of a ‘good trainee’, changing tack on her quest for acceptance:

*And then, I thought there is something wrong here. Something I’ve not done right [choking up]. Like that’s when I mentioned about anaesthetics. Like also a personality issue. But there is was opposite. In the beginning it used to be, but in the end it used to settle down. In fact they used to prefer me over, there were many examples. They used to prefer me over any other anaesthetists. Because that was a personality issue there. So I thought it was a personality issue. What I did was, maybe I’m too senior. I’m not coming across of kind of humble trainee. I’ve got a bit of a kind of a laid back attitude as well. So I thought I might show that I’m learning or something. Ask questions. When I don’t need to ask questions [laughs]. So what I started doing was, I wrote a letter.*

*A referral letter and I said, “Dr K*****, I wrote a letter, a referral letter, Can I show it to you”?*

*He came, he said “leave it on my desk then, when I’ve read it, I’ll let you know”. So, after he read it, towards the end of the day, he came to my room and said, “This is the letter you’ve written. Any damn F1 can write a letter”.*

In this, we see the trainee question herself, and question what is ‘wrong’ with her. In this example, she is questioning how she might position herself differently in the eyes of her supervisor – considering what his expectations of her might be (humble, teachable, motivated, engaged), and taking a step to meet these expectations.

As the listener, I sense the ‘olive branch’ in this example –the action of writing the referral letter representing a move on her part to be positioned differently, accepted as an ‘insider’. And in the trainer’s response, I feel the trainee’s sense of disappointment and further rejection.

**Relationships with Supervisor (s) (Third Reading)**

ST2 supervisor: Feels rejected and inadequate. Relationship breakdown. Defensive voice on the part of the trainee.

**Agency and expectations**: She has particular expectations of her supervisor. These include listening to her, detecting unconscious incompetence, and guiding her. She instead perceives significant monitoring and gatekeeping from her supervisor (which might relate to her vantage point of suspicion). The following except also suggests a feeling of being silenced, and thus misunderstood.

*“The other way I felt I discriminated was the um, in fact, sending to PSU, it should have been addressed by them I feel. I feel they should have addressed it and seen that ok, there was a problem. Golden Minute silence. In the beginning that should have been corrected. Second patient, blood in urine. They should have listened to me, why. I said, but because she was having periods, we know coincidence there. But nobody listens, they just talk over you. So, maybe discussing in detail with the trainee where you were lacking and whether you know it or not. In the tutorials is a good opportunity to discuss that. In tutorials, they did discuss, it was like a rapid fire questions I used to get. They, because they couldn’t pick up anything there, so they just referred to PSU.*

ST3 supervisor: a change of vantage point and experience occurs with a move of practice. See ‘vantage point’. She is moving to a more insider position, and appreciating the world through her supervisor’s eyes.

**I poem- a different perspective**

*I can’t complain*

*I can understand*

*I could send a text*

*I can sit in my room*

*If I was a GP supervisor*

*I would do that*

*I wouldn’t take anything in my hands*

*If I don’t know the trainee*

*I wouldn’t*

*I would see*

*I can’t complain*

**Relationships with wider practice (Third Reading)**

The above example serves as an illustration of the trainee’s cultural position as an ‘outsider’ in her ST2 practice. It appears that she is positioned:

Through a lack of access – to the supervisor and the community of practice (with the ‘door’ as the artefact)

*“after I’ve finished seeing patients I used to keep my door open so that they don’t have to barge through the door. They used to sometimes just barge into the room, “what are you doing still”? Kind of thing.*

*And that made me wonder, because all that’s going on, I used to think, “Are they thinking that I’m watching some movie or something on the computer”. So I used to keep the door open so that they don’t have to just barge in without knocking on the door. So, those kind of things. Once I had left the door open, even then supervisor would pass through and the door opposite was the ST3,*

*She said, “*Amy, have you had your lunch”?*

*[ST3] “Yeah”,*

*[Supervisor] “I want you guys to have your lunch before you see the next lot of patients” and all.*

*But it’s in a loud voice to tell that ‘I’m more concerned about trainees, but not about you”.*

**Societal/cultural messages (Fourth Reading)**

**Artefact – the consulting room door**

Within a GP practice, the doctor works in their own room, and therefore with a door to the room (usually closed when interacting with a patient to protect their confidentiality). Within the narrative, the ‘door’ serves as an artefact to signify the notion of access; both to the supervisor as also to the community of practice. The trainee refers to the ‘door’ in two instances:

1. She is expected to discuss every patient she sees with her supervisor, who is also concurrently in clinic with patients of his own. To gain access to her supervisor (and thus his subsequent feedback, guidance and opportunity for learning), she must wait outside his door until he is available. The closed door in this instance represents the difficulty in gaining access to the supervisor, and emphasizes the feeling of ‘outsider’.

*“Before I was taking my half an hour time and waiting outside their rooms and discussing it, the patient. Then we had another meeting after another month and they said, “You’re still on half an hour and you haven’t done anything about it and your clinical knowledge is poor”.*

*And I said, “Ok, about half an hour I’ll try and do. But I must admit to it, the waiting outside your room sometimes takes 10 minutes and then discussing for another 5 minutes, so 15 minutes you can just put for every patient”.*

*So, they said “no, who said I see patients in 10 minutes. I don’t see patients in more than 10minutes. I see patients very quickly*

*I said you might be, but I still have to wait outside”*

1. The trainee uses to the door (keeping it open) to prevent perceived suspicion from practice staff. In contrast, she perceives that the other trainees in the practice are afforded access and a status of ‘insider’ that she is not – observing these trainees to have access to the supervisors beyond the formal ‘educational’ interaction, and privy to the relaxed corridor and lunchroom culture of the practice. As the listener, I therefore wonder if the ‘door’ in this example serves two purposes for the trainee: firstly to avoid arousing suspicion, but secondly to attempt to gain access to the ‘insider’ position that she perceives afforded to the other trainees:

*“After I’ve finished seeing patients I used to keep my door open so that they don’t have to barge through the door. They used to sometimes just barge into the room, “what are you doing still”? Kind of thing.*

*And that made me wonder, because all that’s going on, I used to think, “Are they thinking that I’m watching some movie or something on the computer”. So I used to keep the door open so that they don’t have to just barge in without knocking on the door. So, those kind of things. Once I had left the door open, even then supervisor would pass through and the door opposite was the ST3,*

*She said, “*Amy, have you had your lunch”?*

*[ST3] “Yeah”,*

*[Supervisor] “I want you guys to have your lunch before you see the next lot of patients” and all.*

*But it’s in a loud voice to tell that ‘I’m more concerned about trainees, but not about you”.*

**The ‘system’**

Within the trainee’s ‘courtroom’ she appears to be defending herself to me (as the researcher). Although most of the narrative discusses her experiences with supervisors and the practice directly, she also alludes to a wider structural influence, and it appears that her defence also relates to this ‘system’ (with myself viewed to be an agent of that ‘system’). This influence is interwoven throughout the narrative, and is a subtle yet consistent presence.

Seema first discusses the ‘system’ as influencing the geographical location of her placements, leading to difficulties in juggling her son’s school commitments with travelling to and from work:

*“There were issues with the other things, nothing to do with supervision. Like I was moving from *Wolverhampton to, I was living in *Wolverhampton. Applied for Birmingham because at Birmingham hospital I was based in *surgery so I was already applying for it. So my son, I made him sit the exam for grammar schools in Birmingham so that we’ll finally relocate there. In between change of mind. Got the GP training, and then I got *Coventry. So the first choice was Birmingham, which I never got.* ***So there was an issue with travelling****.”*

Her training duration, in the form of an extension, was also set by the ‘system’, without discussion, and without her input. Through her use of the phrase ‘an issue I would like to be corrected’, I felt that she may have viewed me as someone who could influence her ‘case’ within the ‘system’:

*“and then I had an extension after that,* ***which was never discussed with me****. This was again an issue* ***I would like to be corrected****. Because it was discussed that this 6 months will not be counted. I had a meeting with *the head of training. This 6 months will never be counted, so I have to do it again.* ***But I didn’t realise****. There will be an extension of 6 months.* ***I didn’t realise*** *it would be equivalent of full time, so it will be more than 6 months. It sinked in afterwards, I think.”*

In various points within the narrative, Seema also refers to the use of documentation (in the form of ‘educator’s notes, made by supervisors within her electronic training portfolio), which is subsequently used as evidence for training decisions by ‘panel’. The ‘panel’ refers to an annual review of a trainee’s progression within their training, and determines if they are competent to proceed to the next stage:

*“For a new trainee, I didn’t know there was an educator note gone on the system until I got called on for the panel. And then they said “there is an educator’s note on your system”*

*So the educator note said, “She’s very unprofessional and she doesn’t have, she’s never punctual. There is an issue with punctuality and she’s very unprofessional and she could do better if she could improve on that”. Something like...And they said, “so you have anything to say about this?”*

*“So they’re trying to find out where I had problems in the past, because they are raising issues now. So they contacted him [the educational supervisor] and said put it on the educators note. So he’s putting the educator’s note in the month of sept about what happened in June*
